# Examining the Relationship Between Screening for Postpartum Depression and Associated Child Health Service Utilization and Costs: A Study Using the All Our Families Cohort and Administrative Data

**DOI:** 10.1007/s10995-023-03831-0

**Published:** 2023-11-08

**Authors:** Shainur Premji, Deborah Ann McNeil, Maria Jose Santana, Eldon Spackman

**Affiliations:** 1 Department of Community Health Sciences, Cumming School of Medicine, University of Calgary, Calgary, AB Canada; 2 Centre for Health Economics, University of York, York, UK; 3 Alberta Health Services, Calgary, AB Canada; 4 Faculty of Nursing, University of Calgary, Calgary, AB Canada; 5 Department of Paediatrics, Cumming School of Medicine, University of Calgary, Calgary, AB Canada

**Keywords:** Postpartum depression, Health service utilization, Costs

## Abstract

**Introduction:**

Despite a recognized association between maternal postpartum depression (PPD) and adverse child health outcomes, evidence examining the relationship between PPD symptoms and associated child health service utilization and costs remains unclear. In addition, there is a paucity of evidence describing the relationship between early identification of maternal PPD and associated health service utilization and costs for children. This study aims to address this gap by describing the secondary associations of screening for maternal PPD and annual health service utilization and costs for children over their first five years of life.

**Methods:**

Mothers and children enrolled in the prospective All Our Families cohort were linked to provincial administrative data in Alberta, Canada. Multivariable generalized linear models were used to estimate the average annual inpatient, outpatient, physician, and total health service utilization and costs from a public health system perspective for children of mothers screened high risk for PPD, low/moderate risk for PPD, or unscreened.

**Results:**

Total mean costs were greatest for children during their first year of life than other years. Those whose mothers were not screened had significantly lower costs compared to those whose mothers were screened low/moderate risk, despite equivalent health service utilization.

**Discussion:**

Findings from this study describe the secondary associations of screening for maternal PPD using a public health system perspective. More research is required to fully understand variations in health costs for children across maternal PPD screening categories.

## Significance

This study describes the relationship between maternal PPD screening status and annual child health service utilization and costs over the first five years of age. Findings from this administrative data study will support decision-makers in understanding the secondary effects associated with maternal PPD screening and inform future cost-effectiveness analyses of PPD screening interventions using a maternal-child health perspective.

## Introduction

Postpartum depression (PPD) affects approximately 13% of childbearing women (O’Hara & Swain, 1996). Symptoms can range in severity, chronicity, and persistence, with onset anywhere between 4 weeks and 12 months postpartum (Chaudron et al., 2004; England et al., 1994; Myers et al., 2013; Netsi et al., 2018). For mothers, PPD is devastating and influences their ability to provide sensitive and responsive caregiving (Myers et al., 2013; Tsivos et al., 2015). Evidence continues to accumulate on the consequences of maternal PPD for children. For example, a 2019 systematic literature review demonstrated an association between PPD and adverse secondary effects for children throughout their lifespan (Moore Simus et al., 2019). At 3 weeks, children affected by PPD are at increased risk of being temperamental and having trouble sleeping (Eastwood et al., 2012), and by 2 years, there are increased risks for delays in communication, emotional, cognitive, and fine motor development (Beck, 1998; Koutra et al., 2013). As children grow older, there is increased risk for emotional, behavioural, and psychosocial difficulties, which influences ongoing school performance and future productivity (Bauer et al., 2015; Hanington et al., 2012; Kingston & Tough, 2014; Korhonen et al., 2012; Valla et al., 2016; Verkuijl et al., 2014). These lifelong effects contribute to high secondary costs of PPD, as reported by Bauer et al. (Bauer et al., 2016). According to their findings, significant lifetime total costs (> £75,000 per woman) are associated with perinatal anxiety and depression, and nearly 60% of these costs are attributed to secondary effects for children (Bauer et al., 2016). For every child exposed to perinatal depression, economic modelling to age 50 demonstrates significant public sector costs, reduced lifetime earnings, and losses in health-related quality of life (Bauer et al., 2015).

Despite a recognized association between PPD and adverse child health outcomes, evidence examining the relationship between PPD symptoms and child health service utilization and costs remains equivocal. For example, a retrospective chart review from an urban United States (US) paediatric primary care clinic indicated no association between PPD and well child visits or acute care utilization over two years postpartum (Kornfeld et al., 2012). Maternal PPD was, however, associated with greater emergency department utilization for children (Kornfeld et al., 2012). In another propensity score-matched US administrative data sample, findings indicated that children exposed to maternal PPD had higher outpatient, physician, emergency department, and early intervention screening utilization over two years relative to children not exposed (Moore Simas et al., 2020). Children of those with PPD also incurred 12% higher total healthcare costs (Moore Simas et al., 2020). A major limitation of these studies was their short time frame of follow-up (two years postpartum), where most children may not have yet had the opportunity to be diagnosed or treated for adverse health outcomes, which typically occurs at ages 3 or later.

In the absence of understanding the relationship between maternal PPD symptoms and child health service utilization and costs, it remains difficult to understand the relationship between interventions to identify and treat maternal PPD and potential secondary effects for the public health system. Yet, this may be a growing concern, given recent attention to evaluating child outcomes specific to interventions for PPD (Forman et al., 2007; Stanley et al., 2004; Stein et al., 2018).

This study aims to examine the association between maternal PPD screening and child health service utilization and costs using longitudinal cohort data linked to provincial administrative data in Alberta, Canada. Previous research demonstrated the effectiveness and cost-effectiveness of the PPD screening pathway over a two-year time horizon (Premji et al., 2019, 2021), where findings suggested the Alberta PPD screening program effectively directed diagnostic and treatment resources to those most in need (Premji et al., 2019). Previous research was limited, however, in that it focused on outcomes for mothers and excluded outcomes for secondary populations, including children (Premji et al., 2021). Yet, this question was raised by decision-makers in Alberta. We therefore turn our attention to examining the relationship between maternal PPD screening and child health outcomes. Findings from this study will support decision-makers in understanding the secondary associations of maternal PPD screening and can be used to inform future cost-effectiveness analyses of PPD screening interventions using a maternal-child health perspective.

## Methods

Ethics approval was obtained from the Conjoint Health Research Ethics Board at the University of Calgary (ID: 190880). A waiver of consent was provided to access child administrative records. Permission to access vital statistics and physician claims data was provided by Alberta Health.

### Sample

Established in 2008, the All Our Families (AOF) prospective pregnancy cohort consists of approximately 3200 mother–child pairs that were recruited during their second trimester of pregnancy (Tough et al., 2017). Several waves of data collection have taken place, focusing on key factors that influence longitudinal maternal and child wellbeing (Tough et al., 2017). Children are currently approaching their adolescent years. For this study, mothers in AOF that consented to providing their provincial health numbers (PHN) were linked to Calgary Zone public health data to obtain information on their date of delivery, PPD screening date and screening score.

### Alberta PPD Screening Pathway

In Alberta, women are screened for PPD using the 10-item self-completed Edinburgh Postnatal Depression Scale (EPDS), provided by public health nurses at their child’s two-month well child public health visit (Alberta Health Services, 2019). The EPDS is a validated and widely used instrument available in multiple languages (Sword et al., 2008) and is often used in research settings to identify women with PPD (e.g., Closa-Monasterolo et al., 2017; Lilja et al., 2012; Netsi et al., 2018). At the time of this study, women with EPDS scores < 10 were considered low risk, scores between 10 and 11 were considered moderate risk, and scores ≥ 12 were considered high risk for PPD. Those screened high risk were referred to their primary care physician for follow-up diagnosis and treatment, per provincial screening guidelines (Alberta Health Services, 2019).

### Data Linkage

**Fig. 1:**
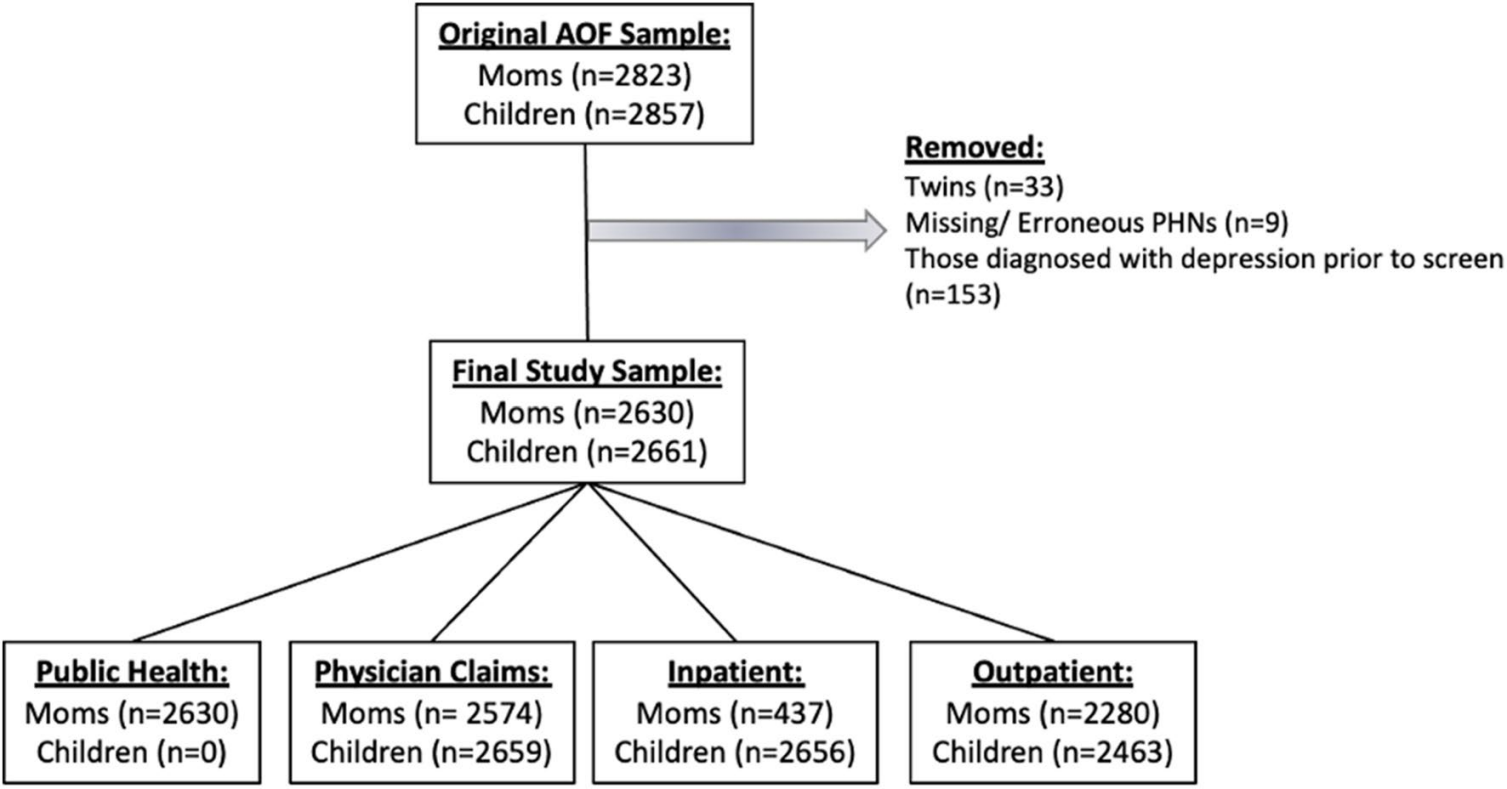
Data linkage

Using vital statistics (births) data, maternal PHN and date of delivery were used to obtain child PHNs. Children were then linked to provincial inpatient, outpatient, and physician claims data. Linkage to administrative data provided information on annual child health service utilization and costs. Following data linkage and cleaning, the final sample for this study consisted of 2630 mothers and 2661 children (Fig. 1).

### Costing

Average costs reflected the annual cost of publicly funded services. All costs were price-adjusted to 2022 Canadian dollars using the Bank of Canada inflation calculator (accessed March 6, 2022). Cost methods align with guidance provided by the Canadian Agency for Drugs and Technologies in Health (Lee et al., 2017).

Inpatient care comprised services provided in hospital for admitted patients, including activities supported by nurses and other professional staff, laboratory and diagnostic services, and the dispensing and administration of drugs (Lee et al., 2017). Inpatient costs were estimated based on a case mix costing approach, which provides estimates on the average case mix group (CMG), including those with similar diagnoses and interventions, and measures resource use in index terms using the resource intensity weight, RIW (Lee et al., 2017). To estimate average inpatient costs, we linked CMG values assigned to each hospital stay to Alberta-specific cost estimates using the 2019 CMG data file sourced from the Interactive Health Data Application (IHDA) provided by the Government of Alberta (Government of Alberta, 2022b).

Outpatient care consisted of patient day visits to a hospital, where patients were not admitted overnight. Visits included diagnostic services, clinic care, telephone consultations, outpatient surgery, or emergency department visits (Lee et al., 2017). Outpatient costs were estimated using the Comprehensive Ambulatory Care Classification System (CACS), which divides cases into ambulatory interventions, emergency department visits, direct diagnostic imaging, rehabilitation, or clinic visits, and estimates direct, indirect, and drug and supply costs for each classification (Lee et al., 2017). Direct costs included nursing services, diagnostic tests, operating and recovery room costs, and indirect costs comprised meals, facilities management, and general hospital operating costs (Lee et al., 2017). Using the 2019 CACS data file sourced from the Government of Alberta IHDA (Government of Alberta, 2022b), costs were matched to CACS codes assigned to outpatient visits for this sample.

Physician services are reported separately from inpatient and outpatient services in Alberta, and include inpatient, outpatient, or community services provided by a medical practitioner, such as a family physician, primary care physician, or specialist (Lee et al., 2017). These costs were estimated based on the Alberta Fee-For-Service (FFS) guidelines and were available for eligible practitioners in the physician claims data, which identified the actual costs paid to physicians for each date of service. Approximately 92% of physician services in our sample were paid using FFS guidelines. The remaining visits took place with a physician enrolled in an Alternate Payment Plan (APP), including practitioners paid by annual salary. Physician costs paid via APP were estimated using shadow billing methods. Common procedure codes available in the physician billing data were used to identify price estimates available in the 2022 February version of the Alberta Schedule of Medical Benefits, provided by the Government of Alberta (Government of Alberta, 2022a). Approximately 90% of APP-related physician services were represented by 30 common procedure codes, with the remaining 10% of services comprising an additional 515 procedure codes. We therefore made the decision to assign shadow price values to the 30 common procedure codes that provided 90% coverage of the APP data.

Total costs were estimated by summing annual inpatient, outpatient, and physician claim costs for children.

### Statistical Analysis

Descriptive analysis took place for sample socio-demographic characteristics, which are reported as means, standard deviations, frequencies, and proportions.

Average annual unadjusted and adjusted all-cause inpatient, outpatient and physician utilization, and average all-cause inpatient, outpatient, physician, and total costs of care were estimated using generalized linear models (GLM). Both cost and utilization data are known to be left-skewed with a substantial number of zeroes (Deb & Norton, 2018) and there is no single dominant method for analysis (Jones et al., 2015). We therefore tested several potential models from the GLM family, and using a linktest and modified Parks test, identified the correct link function and distribution to use. For health service utilization, a GLM model with Gaussian distribution and identity link function appropriately suited the data, and for health costs, a GLM model with Gamma distribution and log link function was best suited. Finally, we conducted post-estimate margins and predictions analyses to derive the average annual health service utilization and costs for children of mothers screened high risk, low/moderate risk, and unscreened. Margins analyses are commonly used in economics to report incremental values in absolute versus relative terms (Onukwugha et al., 2015) and enabled the quantification of differences in service utilization and costs across screening categories. Results report the mean and 95% confidence interval (CI) for health service utilization, and the mean and standard error (SE) for cost data. We further estimated the association between model covariates and incremental health costs using margins analysis and present these results using those unscreened as our reference group, so that the results of this analysis can inform future cost-effectiveness analyses examining the secondary effects of screening versus not screening for maternal PPD.

**Fig. 2:**
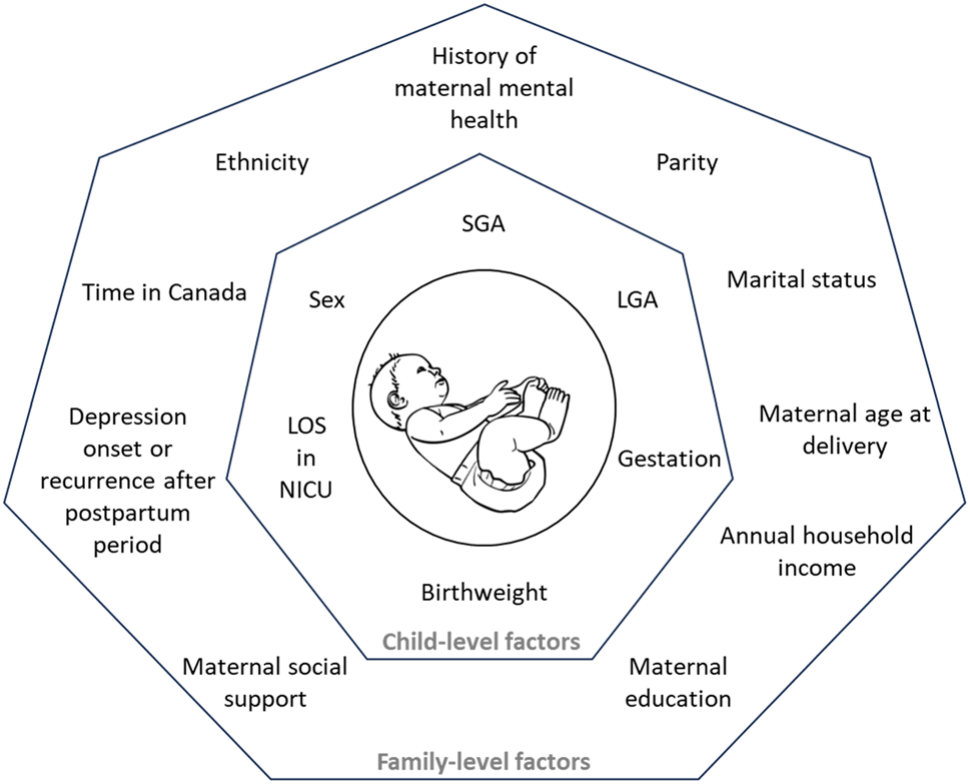
Child and family-level factors considered for this study

All models adjusted for several baseline covariates. For this study, we adopted an ecological perspective, which recognizes that various environmental factors shape human development throughout the lifespan (Bronfenbrenner, 1977). Using a combination of literature review, consultation with maternal and child health experts, and data availability, we shortlisted several child and family-level characteristics that were likely to affect the relationship between postpartum depression and longitudinal child health outcomes (Fig. 2). Using bivariate analyses with a statistical significance set to p < 0.10, we refined our list of covariates to those that demonstrated evidence of an association in our sample. Covariates included in the final GLM models comprised of: total annual household income, child age (birth to 5 years), child sex, child born full- versus pre-term (≥ or < 37 weeks’ gestation), length of stay (LOS) in a neonatal intensive care unit (NICU) within 7 days of birth, child born low versus full birthweight, and whether or not the child was small for gestational age (SGA) at birth. Covariates were sourced from either AOF or administrative data directly. LOS in a NICU, however, was derived using a multistep process. We first identified children with inpatient admissions within 7 days of birth. To determine which of these inpatient stays took place within a NICU setting, we elicited the opinion of a former NICU clinical nurse specialist, who helped identify relevant ICD-10 P and Q codes (“conditions originating the perinatal period” and “congenital malformations, deformations and chromosomal abnormalities”, respectively) that would result in a NICU stay (n = 739). For the remaining children with an inpatient stay within 7 days of birth, we reviewed a combination of their physician claim procedure codes, ICD-10 codes, and total inpatient LOS, and identified a further 8 children with a likely NICU admission. We then generated a new variable for inclusion in this study, which represented the total LOS for children admitted to the NICU within 7 days of birth.

Data linkage, cleaning and analysis took place using Stata 15 (Stata Corp. 2015. Stata Statistical Software: Release 15. College Station, TX, USA: Stata Corp LP). In our data, Year 0 represents the child’s first year of life and Year 5 represent their fifth year of life, up to age six years.

## Results

Mothers in our sample were, on average, 32 years old at the time of delivery, and approximately half were expecting their first child (Table 1). The majority (80%) of mothers were of Caucasian descent, 90% had completed some post-secondary education, and 70% had a total annual household income of ≥ $80,000. Most (95%) were partnered, 90% had lived in Canada more than 5 years, and 93% gave birth when their child was full-term. Nearly 70% of mothers had no previous history of mental health issues. In addition, 53% of children were born male, 95% were born at full birthweight, and 11% were SGA. In our sample, approximately 30% of children were admitted to the NICU within 7 days of birth, with an average LOS of 3.45 days. Overall, 3% of mothers screened high risk for PPD, 84% screened low/moderate risk, and 13% were unscreened.

**Table 1:** Sample characteristics

Variable	Population (n = 2661)*
**Total annual household income**	**N (%)**
< $80,000	757 (29.5)
≥ $80,000	1807 (70.5)
**Maternal education**	
High school or less	275 (10.4)
Greater than high school	2373 (89.6)
**Parity**	
No previous baby	1302 (49.5)
Previous baby	1329 (50.5)
**Ethnicity**	
Caucasian	2082 (78.7)
Other	565 (21.3)
**Marital status**	
Partnered	2526 (95.4)
Not Partnered	122 (4.6)
**Time in Canada**	
Born/Lived > 5 years	2381 (90.3)
≤ 5 years	256 (9.7)
**History of mental health issues**	
No history	1837 (69.4)
History	812 (30.6)
**Child sex**	
Male	1411 (53.0)
Female	1250 (47.0)
**Child gestational age**	
< 37 weeks	169 (6.8)
≥ 37 weeks	2324 (93.2)
**Birthweight**	
Low birthweight (< 2500 g)	128 (4.8)
Full birthweight (≥ 2500 g)	2521 (95.2)
**SGA**	
Yes	281 (10.6)
No	2367 (89.4)
**Child admission to NICU within 7 days of birth**	
Yes	747 (28.2)
No	1902 (71.8)
**Child length of stay in NICU**	**Mean (SD)**
Continuous	3.45 (5.73)
**Maternal age at delivery**	
Continuous	31.65 (4.46)

*Differences between the population n and sub-category frequencies reflect missing responses

*LOS* length of stay, *NICU* neonatal intensive care unit, *SGA* small for gestational age

### Inpatient, Outpatient, and Physician Utilization

**Fig. 3:**
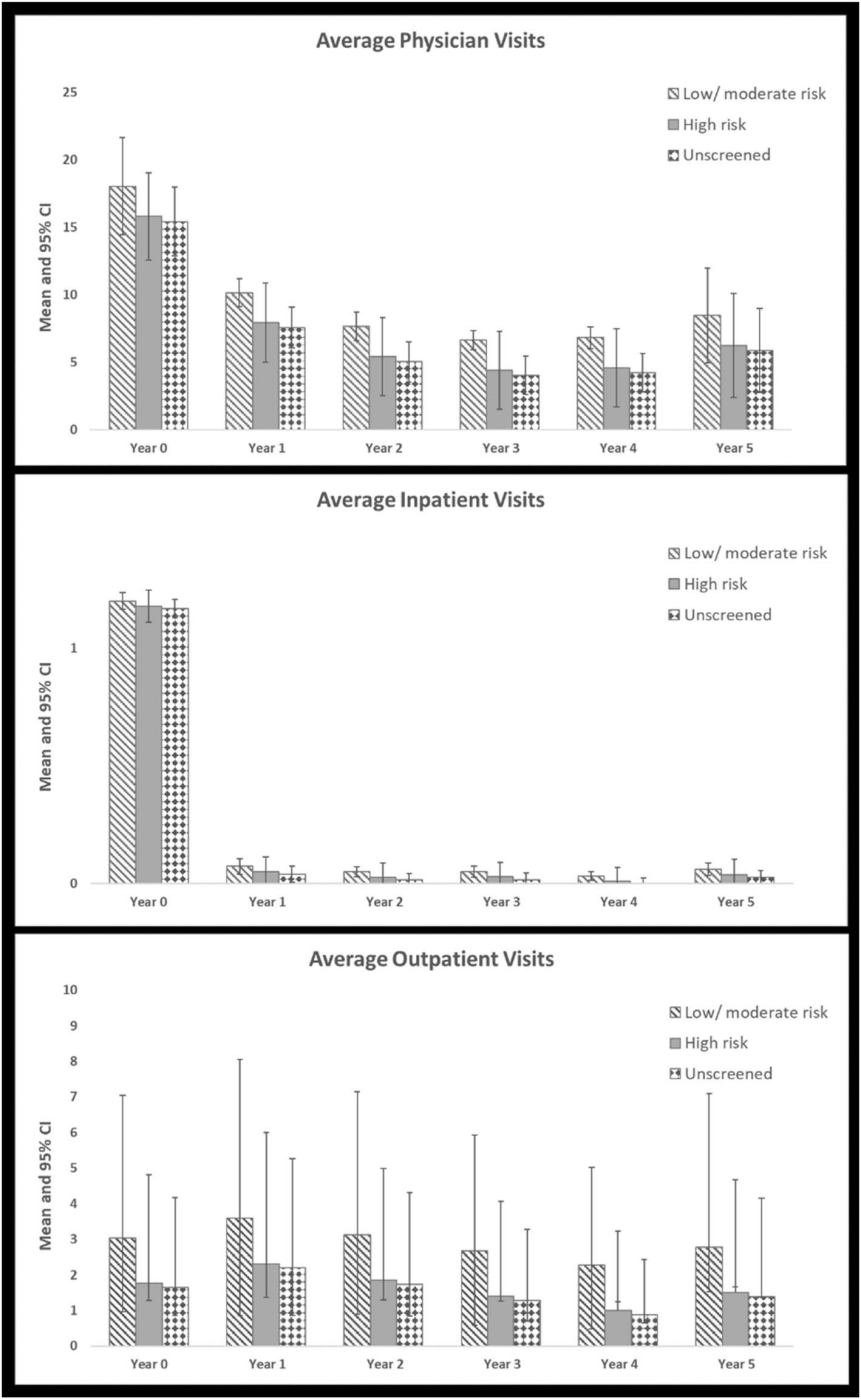
Average annual utilization by PPD screening category and type

In their first year of life, children had, on average, between 15.4 and 18.1 physician visits, 1.2 inpatient stays, and between 1.7 and 3.0 outpatient visits, depending on their mother’s PPD screening status (Fig. 3). Average utilization was not statistically significantly different across screening categories.

When children were between 1 and 5 years of age, annual physician and inpatient utilization across all screening categories fell, averaging between 4.0 and 10.2 for physician visits, and 0 to 0.1 for inpatient stays. Average annual outpatient utilization averaged between 1 and 3.6 visits. Health service utilization for children aged 1 to 5 years were not statistically significantly different across screening categories.

### Inpatient, Outpatient, Physician, and Total Costs

In their first year of life, annual costs attributed to child physician visits spanned approximately $1339-$1691 on average (Table 2). Top reasons for physician visits included routine child health exams (n = 6985), health supervision of the child (n = 2861), delivery of the child (n = 2019), and general symptoms (n = 1299). Child inpatient costs ranged between $14,727 and $43,805 over the first year. Top reasons for inpatient admissions included the delivery of the child (n = 312) and complications related to delivery, such as being low birthweight (n = 152), the presence of neonatal jaundice (n = 88), being born preterm (n = 76), and respiratory failure (n = 64). Child outpatient costs were, on average, between $1,487 and $1,991 in the first year. Top reasons for outpatient visits included developmental screening (n = 158), dietary counseling and service (n = 157), and acute upper respiratory infection (n = 147).

**Table 2:** Annual average costs by screening category and type

		Adjusted total costs		Adjusted physician costs		Adjusted inpatient costs		Adjusted outpatient costs	
		Mean	SE	Mean	SE	Mean	SE	Mean	SE
Unscreened	Year 0	$ 15,556	$ 90,141	$ 1339	$ 1494	$ 14,727	$ 150,063	$ 1487	$ 11,837
	Year 1	$ 3787	$ 21,972	$ 812	$ 918	$ 1171	$ 11,846	$ 1958	$ 15,560
	Year 2	$ 2568	$ 15,002	$ 557	$ 643	$ 475	$ 4787	$ 1587	$ 12,624
	Year 3	$ 2387	$ 13,919	$ 470	$ 547	$ 620	$ 6204	$ 1478	$ 11,734
	Year 4	$ 1660	$ 4,806	$ 469	$ 334	$ 139	$ 715	$ 1008	$ 3687
	Year 5	$ 2115	$ 6303	$ 547	$ 394	$ 411	$ 2196	$ 1047	$ 3920
High risk	Year 0	$ 25,153	$ 145,754	$ 1691	$ 1887	$ 43,805	$ 446,353	$ 1656	$ 13,181
	Year 1	$ 6123	$ 35,528	$ 1025	$ 1160	$ 3482	$ 35,234	$ 2180	$ 17,326
	Year 2	$ 4153	$ 24,258	$ 704	$ 812	$ 1413	$ 14,240	$ 1767	$ 14,057
	Year 3	$ 3860	$ 22,507	$ 594	$ 690	$ 1845	$ 18,453	$ 1646	$ 13,066
	Year 4	$ 2684	$ 7771	$ 592	$ 421	$ 413	$ 2126	$ 1122	$ 4105
	Year 5	$ 3420	$ 10,192	$ 691	$ 498	$ 1224	$ 6531	$ 1166	$ 4365
Low/moderate risk	Year 0	$ 22,475	$ 130,236	$ 1662	$ 1856	$ 36,745	$ 374,407	$ 1991	$ 15,853
	Year 1	$ 5471	$ 31,746	$ 1008	$ 1141	$ 2921	$ 29,555	$ 2622	$ 20,839
	Year 2	$ 3711	$ 21,675	$ 692	$ 799	$ 1185	$ 11,944	$ 2125	$ 16,907
	Year 3	$ 3449	$ 20,110	$ 584	$ 679	$ 1547	$ 15,479	$ 1979	$ 15,716
	Year 4	$ 2398	$ 6944	$ 582	$ 414	$ 346	$ 1783	$ 1350	$ 4938
	Year 5	$ 3055	$ 9107	$ 679	$ 489	$ 1026	$ 5478	$ 1402	$ 5250

*Models adjusted for the following covariates: annual total household income, child sex, gestational age, LOS in a NICU, birthweight, and SGA

*SE* standard error

Total costs within the first year ranged from an average of $15,556 for those whose mothers were unscreened for PPD to an average of $25,153 for those whose mothers screened high risk for PPD (Table 2).

When children were between 1 and 5 years of age, average physician and inpatient costs across all screening categories fell, averaging between $469 and $1025 for physician visits, and between $139 and $3482 for inpatient stays. Average outpatient costs for children rose in year 1 versus year 0, and reduced thereafter. On average, total costs were higher in the child’s first year of life (between $15,556 and $25,153) and fell to year 4 (between $1660 and $2684 in year 4). In year 5, the average total costs rose slightly, ranging between $2115 and $3420.

**Fig. 4:**
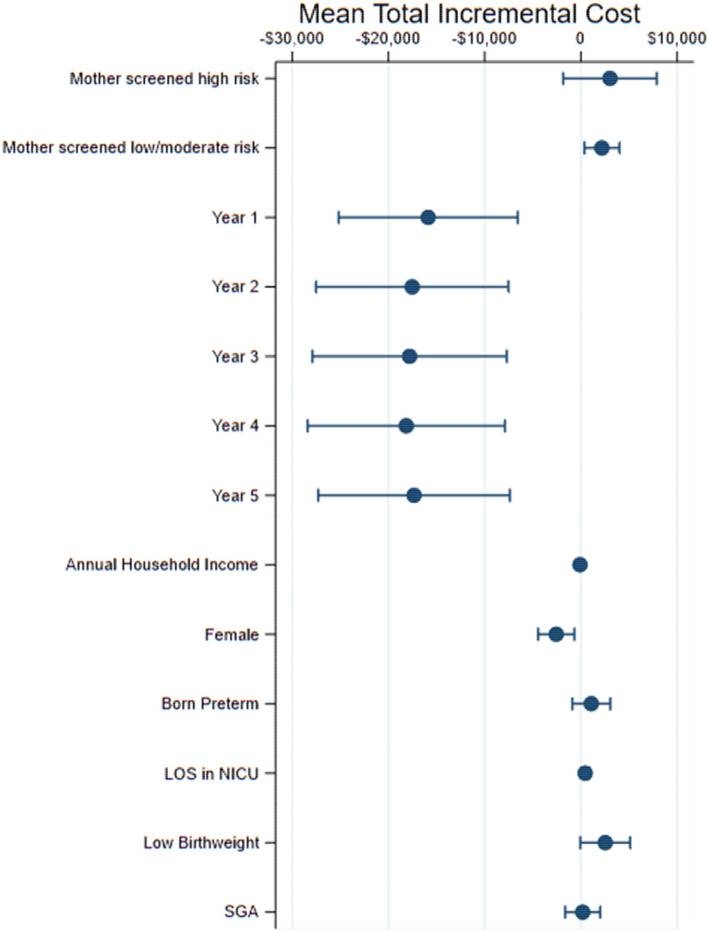
Association between baseline covariates and health costs, with 95% CI

Figure 4 provides the association between baseline covariates and mean total incremental cost. Factors that were statistically significantly associated with total cost included maternal PPD screening category, where children of women screened low/moderate risk had higher mean costs relative to children whose mothers were not screened (p = 0.019), child age (p < 0.001), child sex (p = 0.008), and child LOS in a NICU (p = 0.034).

## Discussion

This study aimed to examine the relationship between maternal PPD screening and longitudinal child health service utilization and costs over five years postpartum. To our knowledge, this is the first study to attempt to understand secondary health service utilization and costs associated with maternal PPD screening interventions in Canada. While study findings indicated that average child health service utilization may be higher for those whose mothers were screened low/moderate risk, these findings were not statistically significantly different from other screening categories (Fig. 3). On the other hand, mean total costs were significantly lower for those whose mothers were unscreened relative to those whose mothers were screened low/moderate risk (Fig. 4), suggesting mean costs were driven by differences in resource intensity between children with mothers across screening categories, where resource intensity was higher for children of mothers unscreened for PPD relative to children of mothers screened low/moderate risk. This remains an area for further investigation, given it is unclear why mothers were unscreened. Socio-demographic characteristics of these mothers suggest they tended to be older and multiparous relative to mothers that were screened (Premji et al., 2019). While studies suggest an association between maternal and child health service utilization, where mothers and children may have similar patterns of service utilization (Minkovitz et al., 2002), our findings suggest there are further nuances to consider, where more research is needed to understand patterns of service use and costs for mothers and children across varying maternal health states.

On average, children in their first year of life had significantly higher total mean costs relative to other years. This finding is consistent with annual total health care expenditures described by the Canadian Institute of Health Information, which reported $819 million total Alberta health care expenditures for children < 1 years in 2019 compared to $508 million for children aged 1–4 years (Canadian Institute for Health Information, 2019).

Notably, all costs beyond the first year of life appeared to progressively decline as children aged, irrespective of maternal screening category (Table 2). In addition, there appeared to be a drop in adjusted inpatient costs at year 4 for children. While these findings were not statistically significant, further research in this area may shed additional insight into these patterns.

While our aim in this study was to describe the relationship between maternal PPD screening category and secondary health service utilization and costs for children, we emphasize that our study findings do not allow for causal interpretations. Rather, they are intended to provide additional insight into possible downstream health system considerations for children of mothers screened for PPD and will support further effectiveness and cost-effectiveness research related to PPD screening programs.

### Strengths and Limitations

There are several strengths and limitations related to this study. Strengths include our ability to link prospective cohort data with multiple provincial administrative databases using unique user identifiers. This allowed us to examine child health service utilization and costs across several settings (e.g., inpatient, outpatient) while adjusting for a rich subset of child and family-level characteristics (Fig. 2). By linking to administrative data, we were also able to examine average service utilization and costs over five years postpartum, which extends previous research in this area and provided additional time for children to be diagnosed and treated for potential adverse health outcomes. Another key strength was our ability to measure PPD symptoms using the EPDS scale. While in our setting the EPDS tool is used to screen and potentially refer women for follow-up diagnosis and treatment, previous research has demonstrated the EPDS is also commonly used in other research settings to identify women with PPD symptoms (e.g., Closa-Monasterolo et al., 2017; Lilja et al., 2012; Netsi et al., 2018).

Prior comparison suggests the baseline socio-demographic characteristics of those mothers who consented, versus did not consent, for their health records to be linked to administrative data were systematically different (Premji et al., 2019). This raises the risk for selection bias towards the null. Another limitation is the potential for non-differential misclassification caused by coding or data entry errors. This likely also resulted in a bias towards the null. Within the broader literature, there is a paucity of research examining the association between screening for PPD and child health service utilization and costs, which created further limitations in that we were unable to compare our results to findings from other settings. Findings from studies that explore the relationship between maternal PPD symptoms and child health service utilization and costs were heterogenous in their unit of output (e.g., utilization and costs were reported over a two-year follow-up period in (Moore Simas et al., 2020), thereby also making comparisons difficult). Further, while a key strength of our study lay in our ability to consider multiple child and family-level factors that could potentially affect the relationship between maternal PPD screening status and child health service utilization and costs, to avoid overfitting our GLM models we only included those variables with a known association in our data into the final analysis. There may, therefore, be a potential for residual (unmeasured) confounding in our study, which also likely resulted in a bias towards the null.

Finally, despite noting a significant association between maternal PPD screening status and annual child health cost, this effect was only noted for those screened low/moderate risk relative to those unscreened. There is a strong possibility that the small sample size of women screened high risk for PPD (n = 77) made it difficult to detect any statistically significant differences for this screening category due to low power.

## Conclusion

Findings from this study describe the secondary associations of screening for maternal PPD and provide additional insight into downstream health service utilization and costs for children. More research is required to fully understand variations in health costs for children across maternal PPD screening categories.

## Data Availability

Data for this study are not publicly available due to ethical restrictions.

## Code Availability

The code that supports the analysis of this study are available on request from the corresponding author, SP.

## References

[CR1] Alberta Health Services. (2019). *Postpartum Depression Screening Guideline* (pp. 1–15).

[CR2] Bauer A, Knapp M, Parsonage M (2016). Lifetime costs of perinatal anxiety and depression. Journal of Affective Disorders.

[CR3] Bauer A, Pawlby S, Plant DT, King D, Pariante CM, Knapp M (2015). Perinatal depression and child development: Exploring the economic consequences from a South London cohort. Psychological Medicine.

[CR4] Beck CT (1998). The effects of postpartum depression on child development: A meta-analysis. Archives of Psychiatric Nursing.

[CR5] Bronfenbrenner U (1977). Toward an experimental ecology of human development. American Psychologist.

[CR6] Canadian Institute for Health Information. (2019). *Table E.1.22.1 Estimate of total provincial/territorial government health expenditures by age and sex in millions of current dollars, by province/territory and Canada*.

[CR7] Chaudron LH, Szilagyi PG, Kitzman HJ, Wadkins HIM, Conwell Y (2004). Detection of postpartum depressive symptoms by screening at well-child visits. Pediatrics.

[CR8] Closa-Monasterolo R, Gispert-Llaurado M, Canals J, Luque V, Zaragoza-Jordana M, Koletzko B, Grote V, Weber M, Gruszfeld D, Szott K, Verduci E, ReDionigi A, Hoyos J, Brasselle G, Escribano Subías J (2017). The effect of postpartum depression and current mental health problems of the mother on child behaviour at eight years. Maternal and Child Health Journal.

[CR9] Deb P, Norton EC (2018). Modeling health care expenditures and use. Annual Review of Public Health.

[CR10] Eastwood J, Jalaludin B, Kemp L, Phung H, Barnett B (2012). Relationship of postnatal depressive symptoms to infant temperament, maternal expectations, social support and other potential risk factors: Findings from a large Australian cross-sectional study. BMC Pregnancy and Childbirth.

[CR11] England S, Ballard C, George S (1994). Chronicity in postnatal depression. European Journal of Psychiatry.

[CR12] Forman DR, O’Hara MW, Stuart S, Gorman LL, Larsen KE, Coy KC (2007). Effective treatment for postpartum depression is not sufficient to improve the developing mother-child relationship. Development and Psychopathology.

[CR13] Government of Alberta. (2022a). *Fees information for health professionals*. February. https://www.alberta.ca/fees-health-professionals.aspx

[CR14] Government of Alberta. (2022b). *Interactive Health Data Application (IHDA)*. http://www.ahw.gov.ab.ca/IHDA_Retrieval/

[CR15] Hanington L, Heron J, Stein A, Ramchandani P (2012). Parental depression and child outcomes–is marital conflict the missing link?. Child: Care, Health and Development.

[CR16] Jones AM, Lomas J, Rice N (2015). Healthcare cost regressions: Going Beyond the mean to estimate the full distribution. Health Economics.

[CR17] Kingston D, Tough S (2014). Prenatal and postnatal maternal mental health and school-age child development: A systematic review. Maternal & Child Health Journal.

[CR18] Korhonen M, Luoma I, Salmelin R, Tamminen T (2012). A longitudinal study of maternal prenatal, postnatal and concurrent depressive symptoms and adolescent well-being. Journal of Affective Disorders.

[CR19] Kornfeld BD, Bair-Merritt MH, Frosch E, Solomon BS (2012). Postpartum depression and intimate partner violence in urban mothers: Co-occurrence and child healthcare utilization. Journal of Pediatrics.

[CR20] Koutra K, Chatzi L, Bagkeris M, Vassilaki M, Bitsios P, Kogevina M (2013). Antenatal and postnatal maternal mental health as determinants of infant neurodevelopment at 18 months of age in a mother-child cohort (Rhea Study) in crete greece. Social Psychiatry and Psychiatric Epidemiology.

[CR21] Lee, K., McCarron, C. E., Bryan, S., Coyle, D., Krahn, M., & McCabe, C. (2017). *Guidelines for the Economic Evaluation of Health Technologies, 4th ed*. CADTH.

[CR22] Lilja G, Edhborg M, Nissen E (2012). Depressive mood in women at child- birth predicts their mood and relationship with infant and partner during the first year postpartum. Scandinavian Journal of Caring Sciences.

[CR23] Minkovitz CS, O’Campo PJ, Chen Y-H, Grason HA (2002). Associations between maternal and child health status and patterns of medical care use. Ambulatory Pediatrics.

[CR24] Moore Simus TA, Huang M-Y, Patton C, Reinhart M, Chawla AJ, Clemson C, Eldar-Lissai A (2019). The humanistic burden of postpartum depression: A systematic literature review. Current Medical Research and Opinion.

[CR25] Moore Simas TA, Huang MY, Packnett ER, Zimmerman NM, Moynihan M, Eldar-Lissai A (2020). Matched cohort study of healthcare resource utilization and costs in young children of mothers with postpartum depression in the United States. Journal of Medical Economics.

[CR26] Myers, E. R., Aubuchon-Endsley, N., Bastian, L. A., Gierisch, J. M., Kemper, A. R., Swamy, G. K., Wald, M. F., McBroom, A. J., Lallinger, K. R., Gray, R. N., Green, C., & Sanders, G. D. (2013). Efficacy and Safety of Screening for Postpartum depression. In *Agency for Healthcare Research and Quality* (Comparative Effectiveness Review, Issue 106, p. 216). Duke Evidence-based Practice Center. 23678510

[CR27] Netsi E, Pearson RM, Murray L, Cooper P, Craske MG, Stein A (2018). Association of persistent and severe postnatal depression with child outcomes. JAMA Psychiatry.

[CR28] O’Hara MW, Swain AM (1996). Rates and risk of postpartum depression—A meta-analysis. International Review of Psychiatry.

[CR29] Onukwugha E, Bergtold J, Jain R (2015). A Primer on marginal effects—Part I: Theory and formulae. PharmacoEconomics.

[CR30] Premji S, McDonald SW, Metcalfe A, Faris P, Quan H, Tough S, McNeil DA (2019). Examining postpartum depression screening effectiveness in well child clinics in Alberta, Canada: A study using the All Our Families cohort and administrative data. Preventive Medicine Reports.

[CR31] Premji S, McDonald SW, McNeil DA, Spackman E (2021). Maximizing maternal health and value for money in postpartum depression screening: A cost-effectiveness analysis using the All Our Families cohort and administrative data in Alberta, Canada. Journal of Affective Disorders.

[CR32] Stanley C, Murray L, Stein A (2004). The effect of postnatal depression on mother-infant interaction, infant response to the Still-face perturbation, and performance on an instrumental learning task. Development and Psychopathology.

[CR33] Stein A, Netsi E, Lawrence PJ, Granger C, Kempton C, Craske MG, Nickless A, Mollison J, Stewart DA, Rapa E, West V, Scerif G, Cooper PJ, Murray L (2018). Mitigating the effect of persistent postnatal depression on child outcomes through an intervention to treat depression and improve parenting: A randomised controlled trial. The Lancet Psychiatry.

[CR34] Sword W, Busser D, Ganann R, McMillan T, Swinton M (2008). Women’s care-seeking experiences after referral for postpartum depression. Qualitative Health Research.

[CR35] Tough SC, McDonald SW, Collisson BA, Graham SA, Kehler H, Kingston D, Benzies K (2017). Cohort profile: The all our babies pregnancy cohort (AOB). International Journal of Epidemiology.

[CR36] Tsivos ZL, Calam R, Sanders MR, Wittkowski A (2015). Interventions for postnatal depression assessing the mother–infant relationship and child developmental outcomes: A systematic review. International Journal of Women’s Health.

[CR37] Valla L, Wentzel-Larsen T, Smith L, Birkeland M, Slinning K (2016). Association between maternal postnatal depressive symptoms and infants’ communication skills: A longitudinal study. Infant Behavior and Development.

[CR38] Verkuijl N, Richter L, Norris S, Stein A, Avan B, Ramchandani P (2014). Postnatal depressive symptoms and child psychological development at 10 years: A prospective study of longitudinal data from the South African Birth to Twenty cohort. The Lancet Psychiatry.

